# Orbital vaso-occlusive crisis mimicking an abscess in pediatric sickle cell disease: A diagnostic pitfall

**DOI:** 10.1016/j.radcr.2025.10.067

**Published:** 2025-11-12

**Authors:** Daniel C. Fong, Wiktoria A. Gocal, Amal Isaiah

**Affiliations:** a Department of Otorhinolaryngology—Head and Neck Surgery, University of Maryland School of Medicine, Baltimore, MD, USA; b Department of Otorhinolaryngology—Head and Neck Surgery, University of Maryland Medical Center, Baltimore, MD, USA; c Department of Diagnostic Radiology and Nuclear Medicine, University of Maryland School of Medicine, Baltimore, MD, USA; d Department of Pediatrics, University of Maryland School of Medicine, Baltimore, MD, USA; e University of Maryland Institute for Health Computing, Bethesda, MD, USA

**Keywords:** Sickle cell disease, Vaso-occlusive crisis, Orbital hematoma, Orbital abscess, Pediatric, Diagnostic pitfall

## Abstract

Sickle cell disease (SCD) is characterized by vaso-occlusive crises (VOC) that typically affect bones with high marrow content. Orbital involvement is rare, observed more frequently in children due to their greater marrow space and rich vascularization. Orbital VOC can clinically and radiologically mimic orbital abscesses, posing significant diagnostic challenges. We present a case of a 6-year-old boy with SCD who developed an orbital VOC initially mimicking an orbital abscess. The patient presented with 4 days of progressive right eye swelling and systemic inflammatory signs. A contrast-enhanced computed tomography demonstrated findings suggestive of subperiosteal abscess, preseptal abscess, subdural empyema, and sinusitis. Subsequent magnetic resonance imaging showed features that could represent either an orbital hematoma or abscess formation. Given the constellation of clinical and radiologic findings suggesting infection with potential intracranial extension, the patient underwent urgent surgical intervention including right craniotomy, right orbitotomy, and nasal endoscopy. Intraoperative drainage revealed dark red-brown fluid without purulence. Surgical pathology demonstrated necrotic fibrovascular tissue with brisk neovascularization, consistent with an organizing hematoma. Cultures remained negative. The final diagnosis was orbital hematoma secondary to vaso-occlusive infarction of orbital bone marrow despite the initial suspicion of an abscess. This case illustrates how orbital VOC in children with SCD can radiologically and clinically mimic an orbital abscess, necessitating a multidisciplinary evaluation to resolve the complicated diagnostic picture. It emphasizes the importance of maintaining a broad differential diagnosis in pediatric SCD patients presenting with orbital findings, particularly when systemic inflammation confounds radiologic interpretation.

## Introduction

Sickle cell disease (SCD) is an autosomal recessive genetic disorder affecting nearly 100,000 individuals in the United States [1], while demonstrating increasing global prevalence [2]. The disease is characterized by rigid, sickle-shaped erythrocytes that impair blood flow through capillaries, leading to vaso-occlusion [3]. Clinically, this presents as acute systemic painful vaso-occlusive crises (VOC) requiring emergent medical care and pain management. Recurrent VOC episodes significantly impact quality of life [4] and are associated with significant morbidity and mortality [5].

VOC most commonly affects bones with high marrow content, including the femur, humerus, and vertebrae. However, VOC can infrequently involve smaller bones, such as those of the orbit [6,7]. Involvement of the orbit is more frequent in children due to their greater marrow space, hypercellularity, and rich vascularization. Due to the confined space within the orbital cavity, an inflammatory response caused by vaso-occlusive infarction may present as rapidly progressive periorbital pain, swelling, and proptosis. Vascular occlusion leads to ischemia and necrosis of orbital bone marrow, with subsequent hemorrhagic transformation resulting in blood extravasation and subperiosteal hematoma formation [[6], [7], [8]].

Orbital involvement following VOC is rare and can mimic other conditions, making diagnosis challenging. The presentation comprising periorbital pain, swelling, and proptosis overlaps infectious etiologies such as orbital cellulitis, subperiosteal abscesses, or even hematologic malignancies such as leukemia. Early recognition of orbital VOC can help optimize pain management and guide appropriate intervention. Given the overlapping presentations, maintaining a broad differential diagnosis is crucial for optimal patient care. While imaging techniques such as magnetic resonance imaging (MRI) and computed tomography (CT) may play an important role in the early detection and management of VOC [9], findings such as subperiosteal hematomas or marrow infarction can be nonspecific, requiring clinicians to correlate with SCD history and other systemic signs.

## Case Report

### Chief Complaint and History

A 6-year-old boy with autism and SCD presented to the emergency department with 4 days of progressive right eye swelling as reported by his caregivers. The patient had a history of multiple prior VOC episodes affecting the long bones but no previous episodes involving the orbit or facial structures. There was no history of recent trauma or insect bites.

### Physical examination

On physical examination, the patient was febrile (temperature 38.9°C) and tachypneic (respiratory rate 32 breaths/minute). Examination revealed significant right periorbital swelling with erythema, proptosis, and restricted extraocular motion. The right eye was displaced inferiorly and laterally. Visual acuity could not be formally assessed due to the patient’s developmental delay and lack of cooperation, but he appeared to track objects with both eyes. Pupils were equal and reactive to light bilaterally.

### Laboratory findings

Laboratory evaluation revealed elevated white blood cell count (15.2 × 109/L) with neutrophilic predominance, elevated reticulocyte count (8.2%), hemoglobin of 7.8 g/dL, and elevated inflammatory markers including C-reactive protein (8.4 mg/dL). Blood cultures were obtained and were pending at the time of surgical intervention. These findings, along with the physical examination findings raised concern for an orbital inflammatory process.

### Imaging studies

**Fig. 1:**
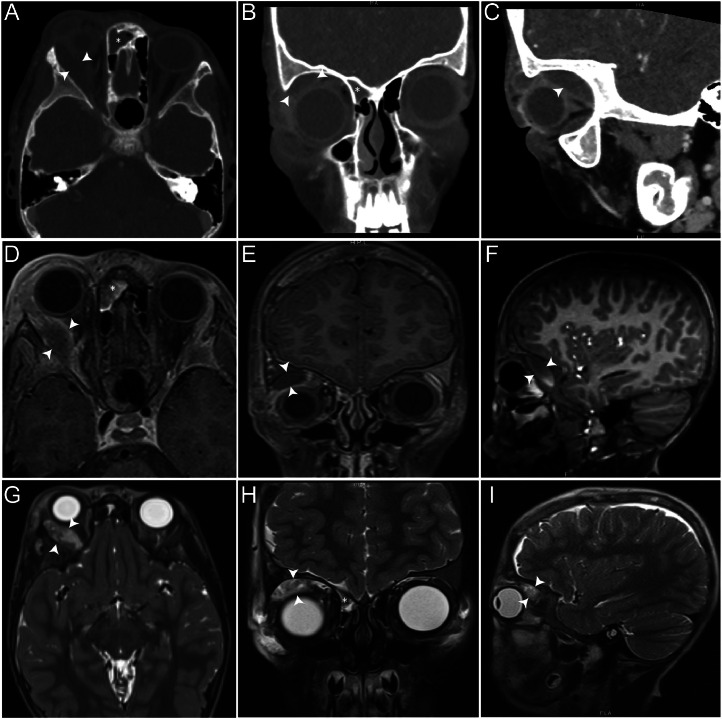
Contrast-enhanced CT and MRI imaging of a child with sickle cell disease presenting with fever, unilateral proptosis, and periorbital swelling. Contrast-enhanced CT of the orbits and paranasal sinuses in axial (A), coronal (B), and sagittal (C) views: White arrowheads in each panel delineate the extent of a right-sided subperiosteal fluid collection measuring ∼1.2 cm in greatest dimension, along the superior and lateral orbital walls, resulting in proptosis and hypoglobus**,** evident in (B)**.** This mass effect causes compression and medial displacement of the extraocular muscles, particularly the superior rectus and lateral rectus muscles. The black asterisk in (B) shows near-complete opacification of the right ethmoid sinus with a corresponding white asterisk indicating the adjacent medial orbital wall and preseptal swelling. The globe is anteriorly displaced and the retrobulbar space is narrow secondary to the posterior extension of the subperiosteal collection. T1-weighted MRI in axial (D), coronal (E), and sagittal (F) views, and T2-weighted counterparts in (G-I). White arrowheads point to the subperiosteal fluid collection, which appears hypo- to isointense on T1 and hyperintense on T2 sequences. The asterisk in (H) indicates the opacified contiguous right ethmoid sinus with air-fluid levels. These T2-weighted images also clearly demonstrate the multiloculated nature of the subperiosteal fluid collection, with internal septations visible in the coronal view (H, white arrowheads)**.** The loculation, while concerning for abscess formation, can also be seen in organizing hematomas as fibrovascular tissue develops within the cavity. The lack of significant soft tissue edema or infiltration further supported hematoma over infectious etiology, though this distinction remained challenging given the clinical context of fever and elevated inflammatory markers.

Given the concern for orbital cellulitis with possible abscess formation in the setting of periorbital swelling and fever, a contrast-enhanced CT scan of the orbits and paranasal sinuses was obtained by the primary pediatric team to evaluate the extent of disease and assess for intracranial extension. This showed a right-sided 1.2 cm subperiosteal fluid collection along the lateral and superior orbital walls with mass effect on extraocular muscles, an 8 × 8 × 14 mm preseptal fluid collection, a 2 mm fluid collection over the right frontal bone, a 5 mm subdural collection, and near-total opacification of the right frontal sinus and patchy opacification of the right anterior ethmoid sinus (Figs. 1A-C). These findings, in combination with fever and elevated inflammatory markers, strongly suggested orbital cellulitis with subperiosteal abscess and intracranial subdural empyema formation, prompting urgent neurosurgical and ophthalmologic consultations.

To better characterize the orbital collection, differentiate hematoma from an abscess based on signal characteristics, and more thoroughly evaluate for additional intracranial complications, a noncontrast MRI of the brain and orbits was recommended by the consulting teams. This (Figs. 1D-I) showed subperiosteal rim-enhancing collections that were T1 hypo- to isointense and T2 hyperintense in the orbits and scalp along with nonspecific T2 hyperintense subdural fluid, and secretions opacifying the right frontal and ethmoid sinuses. The thin rim enhancement was more consistent with a hematoma than an abscess; however, the multiloculated appearance on T2-weighted imaging, combined with sinus opacification and systemic inflammatory signs, prevented definitive exclusion of an abscess from the differential diagnosis.

### Surgical intervention and pathologic findings

Based on the imaging findings and clinical presentation, the leading diagnoses were orbital cellulitis with a subperiosteal abscess and intracranial subdural empyema formation secondary to complicated rhinosinusitis. Given the fever, tachypnea and imaging findings suggesting orbital cellulitis, subperiosteal abscess, right frontal subdural empyema and sinusitis, combined with concern for potential vision-threatening complications, the patient underwent urgent right craniotomy by neurosurgery, a right orbitotomy by ophthalmology, and nasal endoscopy by otolaryngology. Intraoperative drainage of the presumed abscess revealed dark red-brown colored fluid with no purulence. Nasal endoscopy showed no signs of rhinosinusitis and formal endoscopic sinus surgery was deferred. The collected fluid cultures were negative for organisms at 72 hours. Surgical pathology showed necrotic fibrovascular tissue with brisk neovascularization and hemosiderin-laden macrophages, consistent with an organizing hematoma. The absence of purulent material, negative cultures, and characteristic histopathologic findings established the final diagnosis as orbital hematoma secondary to vaso-occlusive infarction of orbital bone marrow.

The orbital hematoma was potentially attributable to a vaso-occlusive infarction of orbital bone marrow with subsequent hemorrhagic transformation leading to subperiosteal hematoma formation, which is a recognized but rare complication of sickle cell disease. This conclusion was supported by the patient’s history of SCD, absence of trauma, negative cultures, and histopathologic findings demonstrating infarction and neovascularization rather than infection.

### Clinical outcome

The patient was managed postoperatively with intravenous fluids, supplemental oxygen, pain control, and prophylactic antibiotics pending final culture results. He showed gradual improvement in periorbital swelling over the subsequent week. Visual function remained intact, and there was no evidence of optic nerve dysfunction. The patient was discharged home on oral antibiotics.

## Discussion

This case underscores how an orbital hematoma secondary to a VOC may closely mimic orbital abscess, particularly when systemic inflammation and sinus opacification are present, potentially leading to misdirected treatment. Clinical presentation of an orbital hematoma in children with SCD may include periorbital swelling, erythema, and tenderness, often accompanied by ocular symptoms such as proptosis, diplopia, and restricted eye movement [6,7,10]. Orbital cellulitis, although a distinct clinical etiology, may present similarly with periorbital swelling, proptosis and pain with eye movements, thus complicating the clinical impression [11]. The most common etiology of periorbital cellulitis is bacterial rhinosinusitis, frequently prompting otolaryngology involvement. If untreated, rhinosinusitis may spread further to the orbit, causing orbital cellulitis, subperiosteal abscesses, orbital abscesses, and cavernous sinus thrombosis [12]. While uncomplicated cases are treated with antibiotics, surgical management is indicated in intracranial extension or abscesses greater than 10 mm [11]. In our case, imaging suggested intracranial extension and a 1.2 cm subperiosteal fluid collection, warranting surgical intervention according to these established criteria.

The clinical and radiological findings in orbital hematomas can overlap significantly with those seen in osteomyelitis and orbital cellulitis, making differentiation challenging [[13], [14], [15]]. MRI is a useful adjunct as infarcts show a clear rim enhancement surrounding the ischemic area, while infectious processes are less well-defined [13]. In this case, the initial CT findings of a subperiosteal fluid collection with proptosis and sinus opacification suggested an infectious process. However, the sinonasal findings were somewhat limited. The MRI therefore provided additional clarity, though not definitive differentiation. In our case, the T1 findings (Figs. 1D-F, white arrowheads) combined with thin rim enhancement suggested a hematoma rather than an abscess. The orbital collection’s T1 hypointense and T2 hyperintense signal characteristics could not completely resolve a hematoma from an abscess. The key distinguishing feature should be the rim enhancement pattern, and the lack of significant surrounding edema or soft tissue stranding which are both more typical of an infarct or hematoma rather than an abscess. Abscesses tend to show a more irregular, thick-walled enhancement with surrounding edema. However, in this case, the fluid collection appeared multiloculated on T2-weighted images (Fig. 1H, white arrowheads). This, along with the child’s elevated inflammatory markers, raised suspicion for an abscess and further confounded the clinical presentation. While MRI features were not strongly supportive of a hematoma (thin rim enhancement), the presence of multiloculation, sinus opacification, fever, and elevated inflammatory markers prevented definitive exclusion of an abscess. Given the potential for vision-threatening complications from both entities and the concern for intracranial extension, surgical exploration was deemed necessary to establish a definitive diagnosis and prevent irreversible complications. These subtle imaging distinctions, when considered alongside the patient’s history of SCD, should raise suspicion for an orbital VOC resulting in hematoma formation, even in the presence of systemic inflammatory signs.

The majority of orbital VOC may be treated with conservative management using oxygen and hydration to reverse the VOC and systemic steroids with antibiotics [14,15]. Prompt recognition and surgical management of orbital VOC are crucial to prevent orbital compression syndrome and vision loss if there is evidence of a large hematoma or optic nerve dysfunction. In cases where imaging and clinical features strongly suggest a hematoma without signs of infection, conservative management may be appropriate. However, when the diagnosis remains uncertain and infection cannot be excluded, as in our case, surgical intervention may be necessary.

Our case highlights the importance of considering different manifestations of VOC in patients with SCD. Initial CT imaging identified fluid collections as potentially representing pre- and postseptal cellulitis with abscess formation, and the patient ultimately underwent an orbitotomy and craniotomy that revealed an orbital hematoma caused by orbital VOC. Orbital infarction is a relatively rare manifestation of VOC with numerous but isolated case reports found in the literature [6,16,17]. This case illustrates a rare orbital manifestation of SCD-related VOC that radiologically and clinically mimicked orbital abscess. It highlights the critical importance of (i) maintaining a broad differential diagnosis in pediatric SCD patients presenting with orbital findings, with VOC-related hematoma included even when imaging and systemic signs suggest infection, (ii) recognizing that systemic inflammation (elevated WBC, fever, CRP) can occur in VOC and does not definitively establish bacterial infection, (iii) understanding that multiloculation on MRI is not pathognomonic for abscess and can be seen in organizing hematomas, and (iv) considering orbital VOC with hematoma formation when imaging demonstrates features atypical for pure infection, especially in patients with known SCD and no clear infectious source.

## Declaration of generative AI and AI-assisted technologies in the writing process

None used during the writing of this paper.

## Patient consent

Complete written informed consent was obtained from the patient for the publication of this study and accompanying images.
